# The acceptance and commitment therapy for smoking cessation in the primary health care setting: a study protocol

**DOI:** 10.1186/s12889-015-1485-z

**Published:** 2015-02-07

**Authors:** Yim Wah Mak, Alice Yuen Loke

**Affiliations:** School of Nursing, The Hong Kong Polytechnic University, Hung Hom, Hong Kong

**Keywords:** Acceptance and commitment therapy, Telephone follow-up, Smoking cessation, Primary health care, Randomized controlled trial (RCT)

## Abstract

**Background:**

Access to effective smoking cessation programs is crucial to reducing smoking-related morbidity and mortality. Several studies have shown promising results for the application of Acceptance and Commitment Therapy (ACT) in managing psychological or behavioral health problems. However, to date, only one study has examined the feasibility of a telephone-based ACT for smoking cessation and it was conducted among a Western population, in the United States. This study reports a protocol for a randomized controlled trial (RCT) examining the feasibility and potential efficacy of an individual, telephone-delivered ACT for smoking cessation in primary healthcare settings among a Chinese population.

**Methods:**

A randomized, two-group design was chosen, with assessment at baseline (before intervention) and via telephone follow-ups at three and six months. Subjects will be proactively recruited from primary healthcare centers. Eligible participants will be randomized to either the intervention (ACT) or control group following the baseline assessment. Both groups will receive self-help materials on smoking cessation. Those in the ACT group will undergo an initial face-to-face session and two telephone ACT sessions at one week and one month following the first session, to be delivered by a counselor based on the treatment protocol. All of the participants will be contacted by telephone for follow-up assessments at three and six months. Treatment fidelity will be assessed by reviewing around one-fifth of audio-recorded telephone calls.

**Discussion:**

To the best of our knowledge, this protocol describes the first RCT of a telephone-based ACT for smoking cessation. It is also the first RCT of ACT for smoking cessation on a Chinese population. The study will provide us with information about the feasibility of a telephone-delivered ACT within a Chinese sample. If effective, this trial will support the development of ACT treatment protocols that could be made available for use by a greater range of clinicians, and offer an evidence base to support alternative treatments for smoking cessation.

**Trial registration:**

ClinicalTrials.gov ID NCT01652508. Registered on 26^th^ July 2012.

## Background

Smoking is the leading preventable cause of death in the United States [1]. The major causes of excess mortality among smokers are diseases related to smoking, including cancers and respiratory and vascular diseases [1]. Quitting smoking before the age of 40 was found to reduce the risk of dying from smoking-related diseases by about 90% [2]. Access to effective smoking cessation programs is therefore, crucial to reducing smoking-related morbidity and mortality.

Despite the development of a wide and broad range of smoking cessation behavioral interventions that have demonstrated considerable effectiveness [3,4], behavioral interventions reach less than 5% of smokers who try to quit [5], indicating that a more accessible means of intervention is needed. Telephone-based interventions make smoking cessation counseling relatively easily accessible in most countries where telephones are common in most households [6]. Telephone-based counseling has also been found to be an effective intervention for smoking cessation in both the West [7,8] and in Asia [9].

According to a study conducted in Hong Kong among parents who smoke, trained counselors from healthcare settings who proactively recruit smokers to participate in interventions are effective at reaching those who have not yet thought of quitting [10]. Studies in the West have also reported that a proactive telephone approach captures pre-contemplative or infrequent smokers who would not initiate cessation counseling on their own [11,12]. These studies imply that proactive telephone-based smoking cessation interventions may be effective and able to reach a higher proportion of people than a standard reactive approach, in which the smoker initiates a call to a smoking cessation hotline.

### Acceptance and commitment therapy for smoking cessation

A recent innovation in behavioral therapy is Acceptance and Commitment Therapy (ACT) [13]. The focus of ACT is on changing one’s relationship with internal experiences rather than directly changing the content of these experiences, so as to enable more adaptive, flexible, and value-based action. The ACT model is comprised of six core processes that work together to increase psychological flexibility including “acceptance”, “defusion”, “self-as-context”, “the present moment”, “values”, and “committed action”. In adopting ACT for smoking cessation, the acceptance component focuses on helping individuals to recognize and accept internal triggers of smoking, including physical sensations, feelings, and thoughts, without trying to control or avoid these experiences (e.g. craving or withdrawal) of smoking. The commitment component emphasizes the articulation of personal values and making the commitment to take action (i.e. to stop smoking) in alignment with these values [13]. It is expected that this will help smokers to respond flexibly to urges to smoke and lead to adaptive changes in behavior.

ACT has a growing empirical base that demonstrates its efficacy for a wide range of disorders. Recent meta-analyses have found ACT to have effect sizes similar to those in traditional Cognitive Behavioral Therapy (CBT) and superior effects to control conditions, including psychiatric disorders, somatic disorders, and stress [14-16]. Several studies have shown promising results for the application of ACT to smoking cessation when delivered face-to-face to individuals, to a group [17-20], through the web [21] or smart-phone applications [22]. Yet there is only one published trial of telephone-based ACT for smoking cessation, despite previous research showing that telephone-based counseling is a cost-effective intervention for smoking cessation [7]. This telephone-delivered ACT intervention was conducted in the U.S. using a single-arm study that included an average of 3.5 sessions of telephone intervention [21]. Among the 14 participants in the study, a quit rate of 29% at 12-month post-treatment was achieved, which doubled the self-reported quit rate of 12% for standard telephone counseling [9,23]. However, it is not clear whether it is feasible and effective to implement this individual, telephone-based ACT for smoking cessation among a Chinese population, in particular, among people in Hong Kong (HK). Therefore, the aim of this study is to examine the feasibility and potential efficacy of telephone-based ACT for smoking cessation using a proactive recruitment approach by conducting a small-scale randomized control trial (RCT) in HK.

## Method

### Design

**Figure 1:**
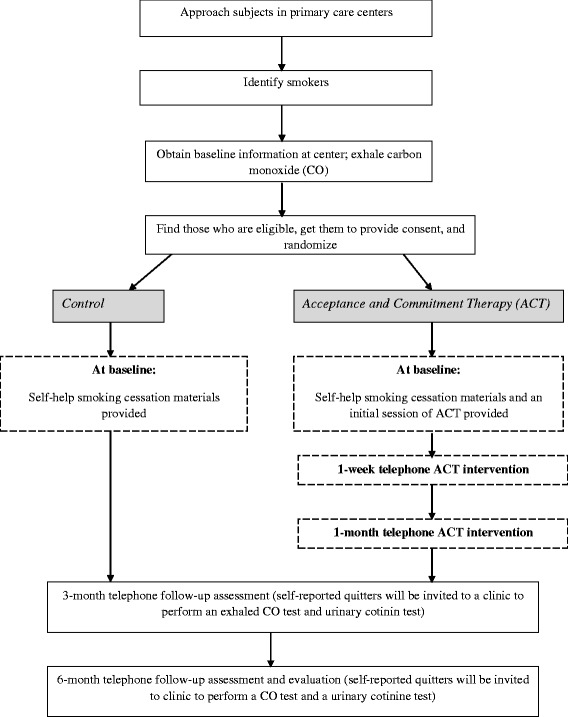
**Study design.**

The study will be a prospective, randomized controlled trial involving an intervention and a control group. The participants will be assessed at three time points (i.e. at baseline, three months, and six months post-intervention). The overall design of the study is illustrated in Figure 1. Ethical approval for the study has been obtained by Human Subjects Ethics Application Review Committee of The Hong Kong Polytechnic University.

### Participants

#### Selection criteria

Individuals who meet the inclusion criteria will be recruited. The criteria are: (1) aged 18 years or above; (2) have smoke at least one cigarette per day in the past 30 days; (3) not currently undergoing any other smoking cessation program; (4) able to communicate in Cantonese; (5) a Hong Kong resident; (6) currently residing in Hong Kong and expecting to continue for the next six months; and (7) have access to a telephone.

#### Sample size

Sample size calculations were based on the primary outcome of smoking cessation- 7-day point prevalence quit rate. As there has been no study that included a brief ACT for smoking cessation in HK, the sample size estimation was taken reference from available ACT studies for smoking cessation [17-20]. Their pooled effect size (Hedge’s g) was 0.475. The comparisons conditions for ACT in these studies included active treatment (e.g. Nicotine Replacement Therapy, Cognitive Behavioral Therapy); Bupropion; and self-help information with longest 12-month follow-up. According to a smoking cessation study conducted in Hong Kong [9], the 7-day point prevalence quit rate of the control group was 7.4% (i.e. receiving self-help materials). With a significance level of 5% and a power of 90%, under the principle of intention-to-treat analysis, 116 participants will be required for the study.

According to a previous smoking cessation study using a proactive approach [10], around 40% of the smokers who were approached completed the baseline interview on smoking patterns and that 45% of them accepted the invitation to participate in a proactive smoking cessation program. Given that attendance in the community services of the six targeted primary health care centers was 13,000 in the past year and that the prevalence of daily smokers was about 11.1% of the population in 2011 [24], it is estimated that recruiting participants from the six health care centers over a period of seven to eight months can support the required sample size of the study.

### Procedure

#### Recruitment

Participants will be recruited from six primary health care centers in HK. Attendees of the center will be approached (Figure 1). Smokers will be identified through a preliminary screening. Individuals who smoke will be invited to complete a baseline questionnaire administered by a member of the clinic’s staff or by a research assistant who conducted the screening. Subjects will also be invited to perform an exhaled carbon monoxide (CO) test in the clinic at baseline. Subjects who meet the criteria for inclusion in the study following the screening and who are interested in participating in the study will be asked to provide their written informed consent.

#### Randomization

Eligible participants will be randomized to either the ACT or the control group. The randomization process will be based on computer-generated randomization numbers placed in sealed and opaque envelopes. A serial number will be given to each envelope. Each envelope will then be opened by the research assistant in sequence, according to the sequence by which the subjects were admitted at the respective center. The randomization procedure will be undertaken by another research assistant not directly involved in the study.

#### Follow-up assessments

At three and six months, the participants will be re-contacted by telephone to complete the follow-up assessments assessing their smoking status, attempts to quit, and ability to deal with physical sensations, emotions, and thoughts without smoking. The assessments will be conducted by trained research assistants who are blind to the treatment condition. Participants who self-report as quitters at the six-month follow-up interview will be invited to perform the CO test in the clinic. Participants in the ACT group will also need to complete an evaluation survey through telephone at the six-month follow up session.

### Intervention

#### ACT

In addition to the self-help materials on smoking cessation given to the participants in both groups, the participants randomized to the ACT condition will be offered an initial individual face-to-face ACT session at the clinic and two telephone follow-up sessions at one week and one month following the initial session of ACT intervention. Each session will be around 15 to 20 minutes, for a total of approximately one hour over the three sessions. The sessions will be delivered by a counselor experienced in delivering ACT. The intervention will be based on a treatment protocol developed in this study, which is guided by previous ACT for smoking cessation protocols [19] and will incorporate all six core processes of ACT. The first session will mainly help the participants to: (1) accept their cravings and withdrawal symptoms as well as to develop skills for dealing with them without smoking, and (2) identify the values involved in quitting and develop a plan to stop smoking at the initial ACT intervention session. Subsequent sessions will include reinforcing the plan that was developed to quit smoking, supporting participants in continued actions to quit, guided by those identified values, and reinforcing the message on the value of quitting. ACT metaphors/exercises will be used throughout the three sessions. Table 1 provides a session-by-session outline of the intervention.

**Table 1:** **Overview of the ACT sessions**

**Session**	**Format**	**Content**
1	Face-to-face	Clarifying values and barriers
		Identifying and clarifying the intention to cease smoking
		Recognizing “creative hopelessness”
		Two kids in the car metaphor
		The ACT in a nutshell metaphor
		Identifying internal triggers
		Appreciating that “control” is always unworkable
		Encourage participants to practice expansion (exercise 1)
		Dropping anchor (exercise 2)
		Introducing and cultivating mindfulness
		Practicing adaptive responses in the presence of internal triggers
2	Telephone	Brief update and bridge from the first face-to-face intervention
		Willingness as an alternative to control (emotional avoidance)
		Emphasize mindful responses with adaptive behaviors
3	Telephone	Brief update and bridge from the last telephone intervention
		Willingness as an alternative to control (emotional avoidance)
		Emphasize mindful responses with adaptive behaviors

#### Control and ACT

At the clinic, the participants will be provided with written self-help materials on smoking cessation (“Tips on quitting smoking”). The printed materials are available to the public and distributed by the Department of Health, HK. The materials describe strategies for tackling cravings and provide information on community resources for quitting smoking.

### Measures

#### Questionnaires

A structured questionnaire will be completed by participants at the baseline, three-month, and six-month follow-up sessions. Demographic data, including age, gender, educational level, current occupation, marital status, and number of people in the household will be recorded at baseline. The questionnaires include questions related to tobacco consumption, such as the number of cigarettes smoked per day, smoking behaviors, withdrawal symptoms, previous attempts to quit, and experiences. The participants’ readiness to quit smoking at different time points will also be recorded [25]. The questionnaires will also include the following scales:

#### Short Form-12 health survey (SF-12)

The SF-12 measures self-perceived general health status [26]. The results are expressed in terms of two meta-scores: the Physical Component Summary (PCS) and the Mental Component Summary (MCS). The PCS and MCS scores have a range of 0 to 100. They were designed to have a mean score of 50 and a standard deviation of 10 in a representative sample of the U.S. population. Thus, scores greater than 50 represent above average health status.

#### Fagerstrom Test for Nicotine Dependence (FTND)

The FTND [27] is a six-item scale that assesses the degree of nicotine dependence. Scores range from 0–10 and are categorized as very low (0–2), low (3–4), medium (5), high (6–7), or very high (8–10). The higher scores reflect higher levels of physiological dependence on nicotine. The FTND has adequate internal consistency and high test–retest reliability [27].

#### Acceptance and action questionnaire-II

The AAQ-II is derived from an earlier version of the AAQ [28], hereafter referred to as the AAQ-I. This is a 10-item self-report measure that assesses the degree of psychological inflexibility and experiential avoidance [29]. Some statements will be modified in relation to the smoking context. For example, the statement “I worry about being unable to control my worries and feelings” will be modified into “I worry about being unable to control my worries and feelings caused by smoking”. Response options will be 1 (strongly disagree), 2 (disagree), 3 (agree), and 4 (strongly agree). Higher total scores on the AAQ-II reflect greater psychological flexibility (and lower engagement in experiential avoidance). The AAQ-II has strong psychometric properties with a mean alpha coefficient of .84. The three-month and twelve-month test-retest reliability has been found to be .81 and .79, respectively [29].

#### Emotion Regulation Questionnaire (ERQ)

The ERQ consists of two subscales related to emotional control, namely cognitive reappraisal and expressive suppression [30]. Response options will be 1 (strongly disagree), 2 (disagree), 3 (agree), and 4 (strongly agree). The higher the total score a respondent obtains in a given subscale, the greater the use of that particular emotion-regulation strategy. Gross and John [30] found a test–retest reliability of 0.69 for both the reappraisal and suppression subscales, and determined that internal consistency of each subscale was acceptable.

#### Process evaluation survey

A process evaluation survey will be given to participants in the ACT group during the six-month follow-up. The survey will examine changes to their ideas of the ACT processes of smoking cessation [20] and their satisfaction with the ACT intervention in helping them to quit smoking.

#### Biochemical measures

The level of exhaled carbon monoxide (CO) will be measured for all participants at baseline by means of a micro smokerlyser CO monitor. Urinary cotinine assays will be collected and the exhaled CO test will again be performed at the six-month follow-up session for self-reported quitters. Breath CO and cotinine have been found to be sensitive methods of determining smoking status [31] and exposure to tobacco smoke [32,33]. Participants with exhaled CO levels of 6 ppm or above [34] or a NicAlert test at level three or above according to the manufacturer will be considered current smokers.

The primary outcome will be self-reported seven-day point prevalence abstinence at the six-month follow up, meaning no smoking at all in the past seven days preceding the six-month follow-up assessment. Secondary outcomes will include: (i) validation of the self-report of not smoking obtained by the CO measurement and urinary cotinine; (ii) readiness to quit smoking; (iii) average number of cigarettes consumed daily in the past seven days; (iv) number of attempts to quit in the past month; (v) self-report of not smoking in the past seven days; and (vi) household smoking status in the past month.

### Treatment feasibility

The feasibility of the ACT program will be assessed by investigating: (i) program acceptability as measured by the participants’ and facilitators’ perceptions of the program; (ii) participant recruitment; (iii) program attendance and retention; and (iv) the participants’ acceptance of physical cravings, emotions, and thoughts related to smoking, as well as their commitment to quit smoking.

### Treatment fidelity

Standardization in the delivery of the intervention will be enabled by employing a written manual for ACT. Treatment sessions will be audio-recorded and random samples of audio files (15-20%) will be rated by two trained independent assessors for treatment fidelity using The ACT Core Competency Rating form [28]. The intraclass correlation will be computed for ratings between the two assessors to ensure that the treatment fidelity ratings are reliable. Feedback on the outcome of these reviews will be provided to the research team and subsequently explored under weekly supervision.

### Statistical analysis

Statistical analyses will be carried out using the Statistical Package for Social Sciences (SPSS) Statistics v.21 software program. Statistical significance will be considered as p < .05. Descriptive statistics for basic demographics, number of sessions attended by the participants, intervention total length, and per-call duration will be computed. Baseline data will be analyzed to check the comparability of the intervention and control group. The primary analysis is differential changes in the ACT group versus the control group from the baseline to the three-month and six-month follow-up sessions, which will be analyzed within the framework of the Treatment Group. Analyses will be undertaken using mixed-model repeated measures. Primary evidence of the efficacy of ACT will be a significant two-way interaction demonstrating greater change in outcome measures in the ACT group from pre- to post-intervention. An intention-to-treat analysis will be performed. This approach is regarded as the most appropriate method of analyzing RCT data by the highest-quality journals, which have adopted the CONSORT standard [35].

## Discussion

While research has found that ACT is effective for smoking cessation, no controlled trial studies of telephone-based ACT for smoking cessation has been conducted to date. To the best of our knowledge, this will be the first RCT examining the effectiveness of telephone-based ACT for smoking cessation. It is also the first study that examines the feasibility of the use of telephone-based ACT among a Chinese population.

The results of this study will provide clinicians with the necessary information to develop strategies to help smokers to stop smoking by using a less intense intervention at a time convenient to them. If found to be effective, this therapy can potentially be practiced by a wider range of clinicians than other therapies, given that it is much briefer and readily manualized than existing psychological treatments. It also has the potential to serve as an alternative useful tool for those who do not benefit from other commonly used treatments (e.g. Nicotine-Replacement Therapy, Cognitive Behavioral Therapy). In addition, this proactive recruitment approach may reach service users who might not otherwise be reached using traditional reactive approaches (e.g. Quitline, smoking cessation clinic, etc.). Information on the feasibility of this innovative smoking cessation intervention will provide further insight into its development. If this intervention program is well received by smokers and effective in helping those who smoke to quit, it could be offered to a larger population in Hong Kong or elsewhere at a low cost. It is believed that this telephone-based ACT will, in particular, acceptable to people in HK, as it can save time and allow them time for competing commitments. One anticipated difficulty for the study is subject requirement. However, the plan to establish multiple venues for recruitment with the inclusion of six primary care centers and a longer recruitment period should go some way towards ensuring an adequate participant pool.

## Abbreviations

AAQ-II: Acceptance and action questionnaire-II
ACT: Acceptance and commitment therapy
CO: Carbon monoxide
ERQ: Emotion regulation questionnaire
FTND: Fagerstrom test for nicotine dependence
HK: Hong Kong
MCS: Mental component summary
RCT: Randomized controlled trial
SF-12: Short form-12 Health survey
SPSS: Statistical package for social sciences
TCM: Traditional Chinese medicine
